# Published evidence: How good?

**DOI:** 10.4103/0970-1591.44243

**Published:** 2008

**Authors:** Nitin S. Kekre

**Affiliations:** Department of Urology, Christian Medical College, Vellore - 632 004, Tamilnadu, India. E-mail: editor@indianjurol.com

It has been our endeavor to sensitize all the readers of Indian Journal of Urology toward the significance of evidence-based medicine. In the current and the last issue, Philipp Dahm *et al* have written two wonderful articles about what every surgeon should know about surgical trials. They have emphasized that it is of paramount importance to weigh the quality of published literature. But unfortunately, it is not uncommon for us to find an article which is supposed to be of good quality with good statistical analysis being refuted by another article or meta analysis.

Why does this happen? John PA Loannidis, an epidemiologist from University of Loannina School of Medicine, Greece, in his essay titled “Why most published research findings are false” has raised very pertinent and serious question over the value and quality of published research. He points out that the chance of a research claim being true depends upon study power, bias number of other studies on the same question, and more importantly, the ratio of true to no relationships among relationships probed in each scientific field. Even this does not spare the so-called gold standard RCT's. This is because of greater flexibility in design, definitions, outcomes, and analytical modes. One should not underestimate the greater financial interests (Industry funded research) and author's prejudices. So what should be the direction of research in medicine? According to Loannidig, we must acknowledge the defects and reform the research strategy. I would strongly recommend all of you who are interested in this field to read his essay. loannidis JPA (2005). [Why most published research findings are false. PLoS Med 2 (8): c124]

This issue contains a symposium on “Retrograde intrarenal surgery: Where are we today?” which appears to be the current fashion of endourology. Dr. Percy Chibber and his co-authors have done a fabulous job of putting this in correct perspective and would help the practicing urologists to decide the place of retrograde intrarenal surgery in their clinical practice. I thank Dr. Percy Chibber and the co-authors for their contribution.

This issue also carries an article on Prof. HS Bhat by Dr. Ganesh Gopalakrishnan. He truly was the Doyen of Indian Urology and continues to serve the humanity till date. I consider myself privileged of having an opportunity to interact with him during my visit to SSSIHMS, Puttaparthy.

The year 2008 had been a fascinating one mixed with highs and lows. In this year, the world witnessed melting down of the biggest names in world of business. India achieved a distinction of sending space vehicle on the moon and an Afro-American became the President of USA. I hope and pray that 2009 would bring in peace and prosperity. I would like to take this opportunity to thank colleagues of the editorial board and Medknow Publications for their hard work.

Wish you all a very happy 2009.

